# A multiscale analytical approach to evaluate osseointegration

**DOI:** 10.1007/s10856-018-6068-y

**Published:** 2018-05-07

**Authors:** Anders Palmquist

**Affiliations:** Department of Biomaterials, Sahlgrenska Academy at University of Gothenburg, Göteborg, Sweden

## Abstract

Osseointegrated implants are frequently used in reconstructive surgery, both in the dental and orthopedic field, restoring physical function and improving the quality of life for the patients. The bone anchorage is typically evaluated at micrometer resolution, while bone tissue is a dynamic composite material composed of nanoscale collagen fibrils and apatite crystals, with defined hierarchical levels at different length scales. In order to understand the bone formation and the ultrastructure of the interfacial tissue, analytical strategies needs to be implemented enabling multiscale and multimodal analyses of the intact interface. This paper describes a sample preparation route for successive analyses allowing assessment of the different hierarchical levels of interest, going from macro to nano scale and could be implemented on single samples. Examples of resulting analyses of different techniques on one type of implant surface is given, with emphasis on correlating the length scale between the different techniques. The bone-implant interface shows an intimate contact between mineralized collagen bundles and the outermost surface of the oxide layer, while bone mineral is found in the nanoscale surface features creating a functionally graded interface. Osteocytes exhibit a direct contact with the implant surface via canaliculi that house their dendritic processes. Blood vessels are frequently found in close proximity to the implant surface either within the mineralized bone matrix or at regions of remodeling.

## Introduction

The term osseointegration, meaning a direct structural connection between living bone and an implant surface, was coined by Professor P.I. Brånemark [1]. The pioneering development of the osseointegrated dental implant during the 60 and 70s [2] led to their global introduction in the 80s and is currently a routine treatment modality in dentistry. Since then, other clinical applications have emerged such as osseointegrated facial prostheses, bone anchored hearing aid, and major limb amputation prosthesis [3].

The healing around commercially pure titanium (cp-Ti) implants was originally described as being very similar to natural bone healing [4], hence being biocompatible and avoiding the formation of fibrous encapsulation. Transmission electron microscopy (TEM) was used extensively, and demonstrated intimate contact between bone and the implant surface, however, often separated by an electron lucent or electron dense layer in the range of 20–50 nm, followed by mineralized collagenous bone, despite the frequent use of demineralization (summarized in [5]). An alternative theory, especially for roughened implant surfaces was the contact osteogenesis theory [6], where a µm thick cement line is formed at the immediate implant surface [7], similar to the cement line separating osteons of different ages, characterized as a collagen deficient hypermineralized zone [8].

Recently, it has been proposed that bone healing around implants is a “foreign body reaction in equilibrium” with a bony encapsulation, characterized as a poorly vascularized dense tissue interfacing the implant surface [9]. However, no experimental data has been shown to support this latter theory and standardized protocols for the evaluation of osseointegration at different length-scales and resolutions are needed in order to characterize the bone tissue interfacing an implant.

The aim of this article is to describe an analytical strategy to probe the different length-scales of osseointegration going from macro to nano. The focus is to have a platform enabling comprehensive, multiscale and multimodal analysis of single samples, allowing detailed characterization of clinical retrieved samples where the limited amount of material poses a major challenge. This review is limited to structural and compositional analyses and does not cover the cellular and molecular biology underlying osseointegration, which may be found elsewhere [10–14].

## Bone tissue

**Fig. 1:**
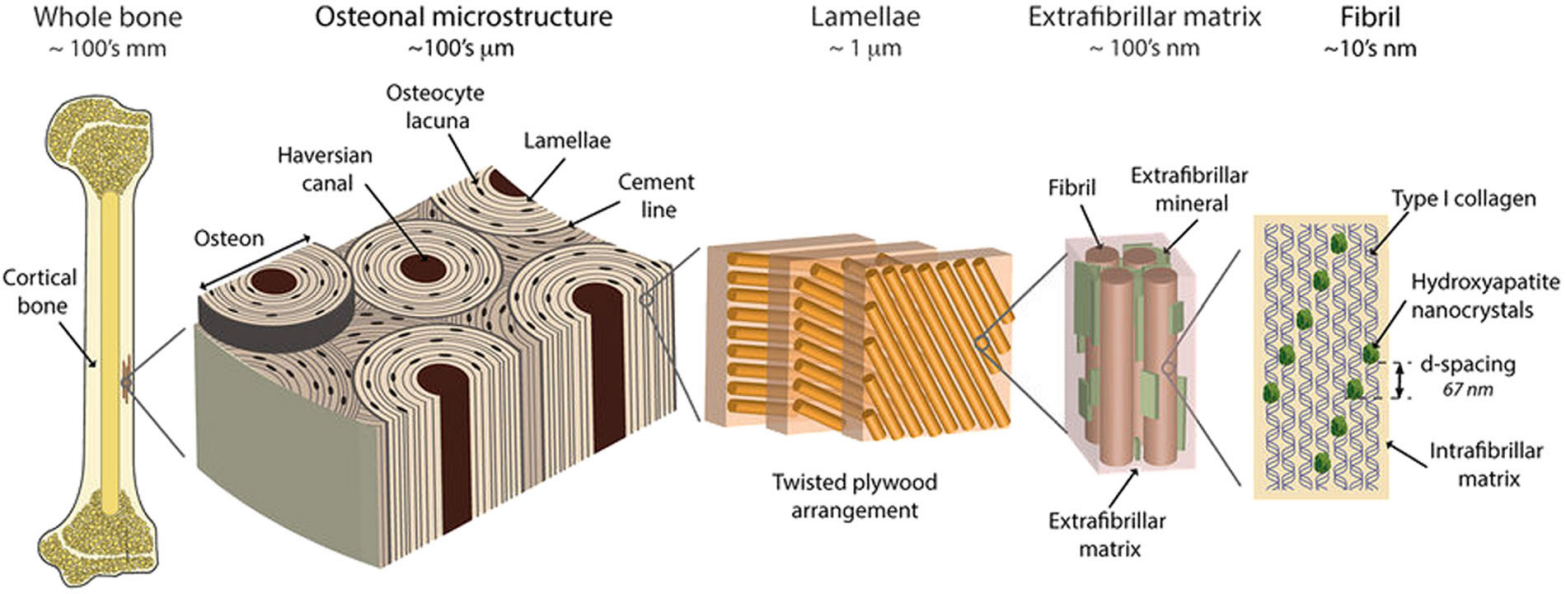
Bone hierarchy: Illustration of the different hierarchical levels of bone tissue with corresponding length scales, from macro to nano scale. Reprinted with permission from Springer Nature [71]

Bone tissue, a highly hierarchical biocomposite material (Fig. 1) mainly composed of an organic matrix (collagen type I) and an inorganic reinforcement phase (apatite), is an adaptive tissue constantly undergoing remodeling.

Bone formation occurs through secretion of tropocollagen by the osteoblasts (bone forming cells) which is later mineralized. The early healing is characterized by the rapid formation of woven bone, an unorganized bone tissue which is later remodeled into more ordered bone through the coupled action of osteoclasts (bone resorbing cells) and osteoblasts. In this process, the osteoblasts start a coordinated action of bone formation and it is believed that they can orient the collagen matrix [15] leading to the higher hierarchical levels through a bottom-up approach. In the bone formation process, some osteoblasts stop producing extracellular matrix and instead start undergoing differentiation into osteocytes and become entrapped within the matrix produced by their neighboring osteoblasts. The osteocyte resides within lacunae (ellipsoidal shaped cavities in the bone) and the osteocyte is connected with the neighboring osteocytes, the blood circulation, and to the osteoblast (in a bone formation site) and lining cells (in a steady state) via the lacuno-canalicular network (LCN). It is believed that the osteocyte is the mechanosensing cell [16] responsible for adaptive remodeling of bone, hence maintenance of structural integrity and long-term performance.

The mechanics of bone (in the context of bone formation around metal implants, mechanical load-transfer from the metal into the surrounding bone, and adaptive remodeling etc.) are strongly dependent on the nanoscale organization and composition. It is therefore important to understand how bone forms on an implant surface, generally, and whether this nanoscale organization varies with respect to implant surface modification. Technical challenges in the preparation of TEM samples of the interfacial bone led to the development of strategies involving elimination of the bulk properties of the implant in the sample preparation. This was achieved either through the use of polymer implants with a thin coating of titanium (restricted to experimental studies) [17], mechanically separating the implant from the embedded bone tissue [18], or by electrochemically dissolving bulk titanium while the surface oxide layer was left intact [19]. All these techniques had their shortcomings and the focused ion beam (FIB) milling technique for the preparation of intact bone-metal interface samples for TEM was introduced in 2006 [20].

## Ex vivo tissue preservation and handling

**Fig. 2:**
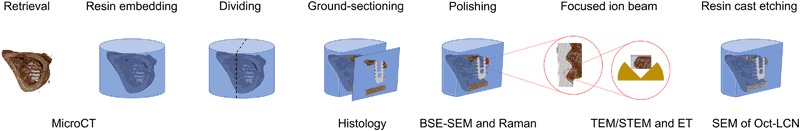
Multiscale and multimodal analysis: The work-flow from retrieval and sample processing to sequential evaluation using a range of imaging and complementary spectroscopic techniques allowing comprehensive analysis of the same specimen

Tissue-implant samples are retrieved from the biological milieu, either from designed experimental models in animals or clinically retrieved from patients. The tissues undergo chemical fixation by immersion in aldehyde solutions, dehydration in a graded series of ethanol, and successive resin infiltrated prior to polymerization. This procedure was developed in order to be able to prepare undecalcified ground-sections for histology [21]. Fixation, dehydration, and resin embedding tend to induce dimensional changes and distort the ultrastructure [22, 23]. However, it is possible to perform high-resolution analyses in combination with histology [24], and therefore such sample preparation methods are suitable for a successive analytical strategy even for clinically retrieved implants where limited volume of biological tissue and/or number of samples are obtained. In Fig. 2, the sample preparation chain is shown to illustrate the correlative approach enabling multiscale and multimodal analysis of the intact bone-implant interface.

## Successive analytical approach and examples

To exemplify the successive analytical approach, examples are shown of a hierarchically structured implant surface having a distinct microtopography and superimposed nanostructure (Supplementary Figure 1), from several experimental and clinical retrieval studies [24–30] in combination with the development of different analytical techniques adapted for the bone-implant interface [30–34].

### Micro-focused X-ray computed tomography (micro-CT)

**Fig. 3:**
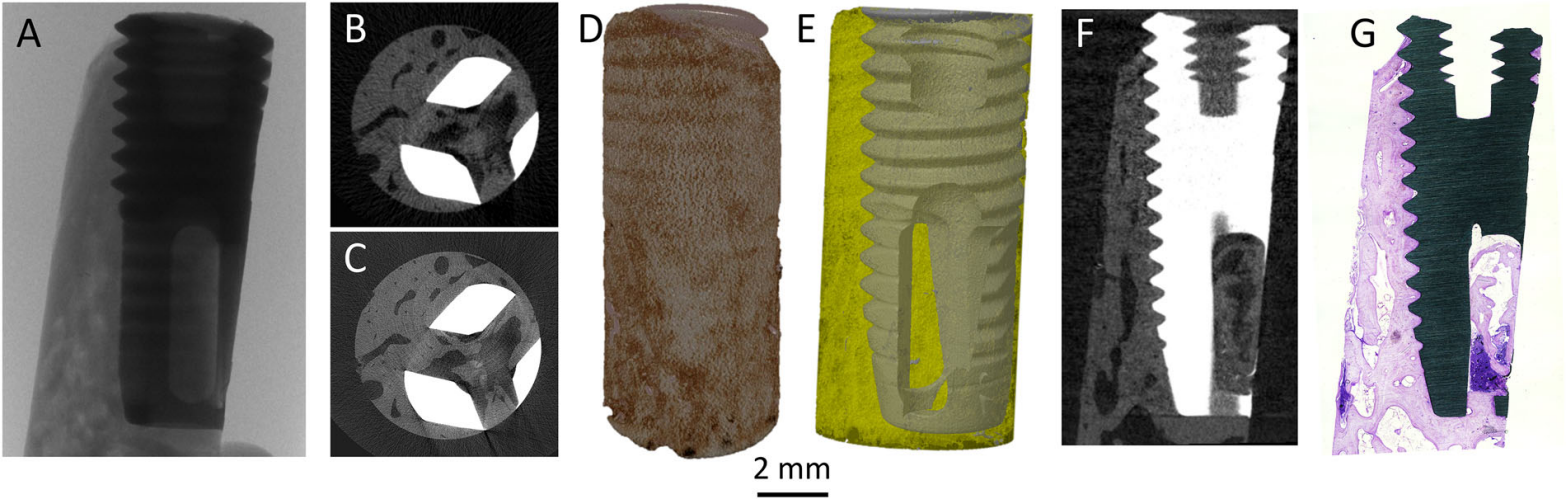
Macro level of osseointegration: Different views of a micro-CT analysis starting with **a** X-ray imaging. **b** Typical reconstruction of a low-resolution scan, 12 µm voxel size (typically 15 min). **c** Typical reconstruction of a high-resolution scan, 2.5 µm voxel size (typically 8 h). **d** A three-dimensional volume rendering of the reconstructed image stack. **e** A three-dimensional surface rendering of the segmented data set representing the implant (grey) and bone (yellow) which has been made semi-transparent. **f** Data set rotated to locate the slice obtained as the histological ground-section. **g** Overview image of the ground-section stained by toluidine blue

Micro-CT is the least destructive technique, where minimal sample preparation is needed and is emerging in the field of osseointegration to study the bone-implant interface. The technique offers a rapid data acquisition, generating results without the need for tedious sample preparation that is generally necessary for histology. This means that samples can be scanned at any stage from the time of retrieval to post-embedment. The biggest advantage of micro-CT over histomorphometry is that morphometric measurements can be performed in 3D rather than having one *representative* histological section from each sample. Furthermore, micro-CT imaging can be directly correlated to histology by locating the histological section in the micro-CT data set (Fig. 3).

Nevertheless, the technique is associated with artifacts introduced during data acquisition, many of which may be corrected for during reconstruction. However, widely differing densities of the metal implant and the biological tissue compromise the analysis close to the implant surface [35, 36]. The technique has been validated to assess bone volume accurately by correlating with histological measurements [37, 38] even at fast/low resolution scans (e.g., 14 µm). While some investigators have reported a correlation between micro-CT and BSE-SEM based measurements of bone-implant contact [39], it is advisable that measurements be correlated with histology and histomorphometry.

### Light optical microscopy (LM)

The *gold standard* in biomaterials research is to evaluate tissue response to biomaterials using simple optical microscopy, where both quantitative histomorphometry and qualitative histology can be performed. Qualitative histology enables a detailed description of the state of the tissues, the maturity (woven, lamellar, or osteonal) and the presence of cells such as different bone cells (osteoblasts, osteoclasts and osteocytes), adipose cells, blood cells, inflammatory cells and multinucleated giant cells as well as the general morphology and to some extent the structure and alignment of the tissues. A limiting factor is the sample preparation, where a section of single-cell thickness is preferable. Moreover, the fixation and dehydration steps must be optimized in order to avoid large artifacts from shrinkage especially in unmineralized areas, i.e., the marrow compartment. It has been shown that quantification of bone-implant contact is highly dependent on the sectioning direction as well as the thickness of the ground-section [40, 41].

**Fig. 4:**
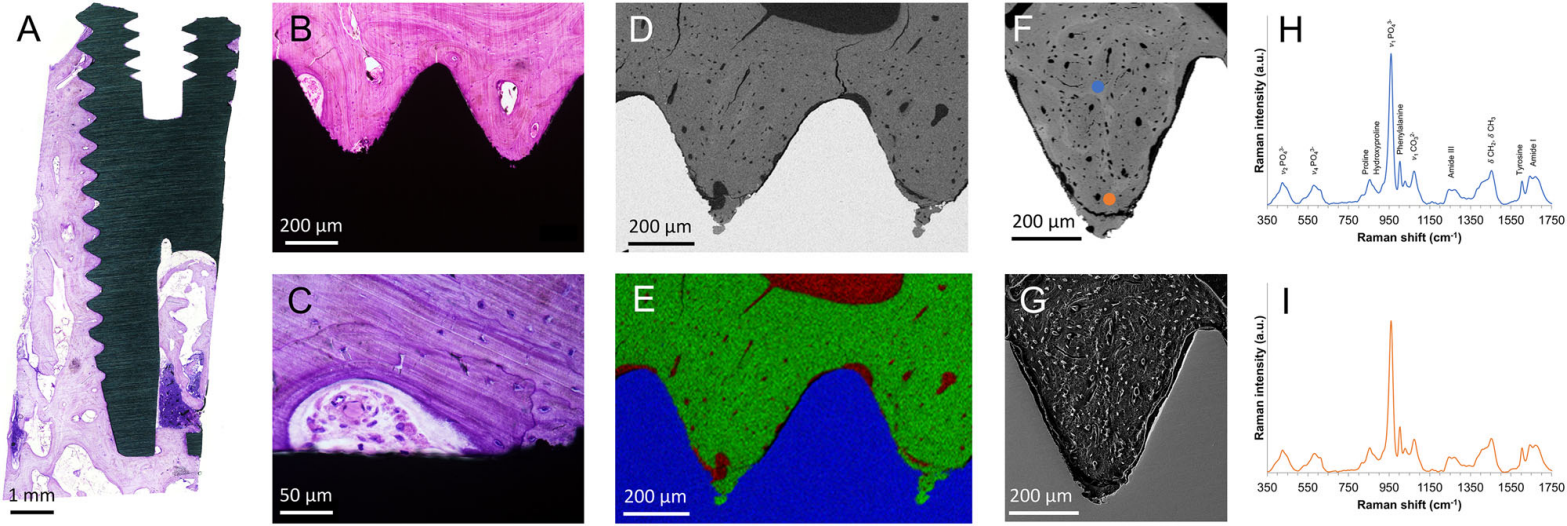
Microscale osseointegration: Light optical microscopy combined with BSE-SEM, EDS, Raman spectroscopy and SE-SEM of resin cast etched samples, showing the successive increase in magnification to follow the bone growth adjacent to the implant. **a** Overview histological image, showing the amount of bone tissue around the implant. **b** Closer view of two threads almost completely filled with mature bone tissue. **c** A closer view at the implant surface showing a remodeling zone with active bone formation, blood supply. Osteocytes are visible in the bone with stained nuclei. **d** BSE-SEM image of two threads showing a similar picture as the histology, mature bone filling the threads. **e** Correlative elemental mapping of the two threads, showing calcium (green), titanium (blue) and carbon (red). **f** Single thread in BSE-SEM showing a biomechanical testing induced fractured zone at the thread valley as well as osteocytes and blood vessels throughout the tissue. **g** Corresponding thread after resin cast etching, showing the plastic surrounding osteocytes and blood vessels protruding from the etched bone surface. **h** and **i** Raman spectra from the corresponding spots marked in **f**, showing the molecular composition of the tissue. Images modified and reprinted with permission from John Wiley & Sons and Public Library of Science [25, 27]

**Fig. 5:**
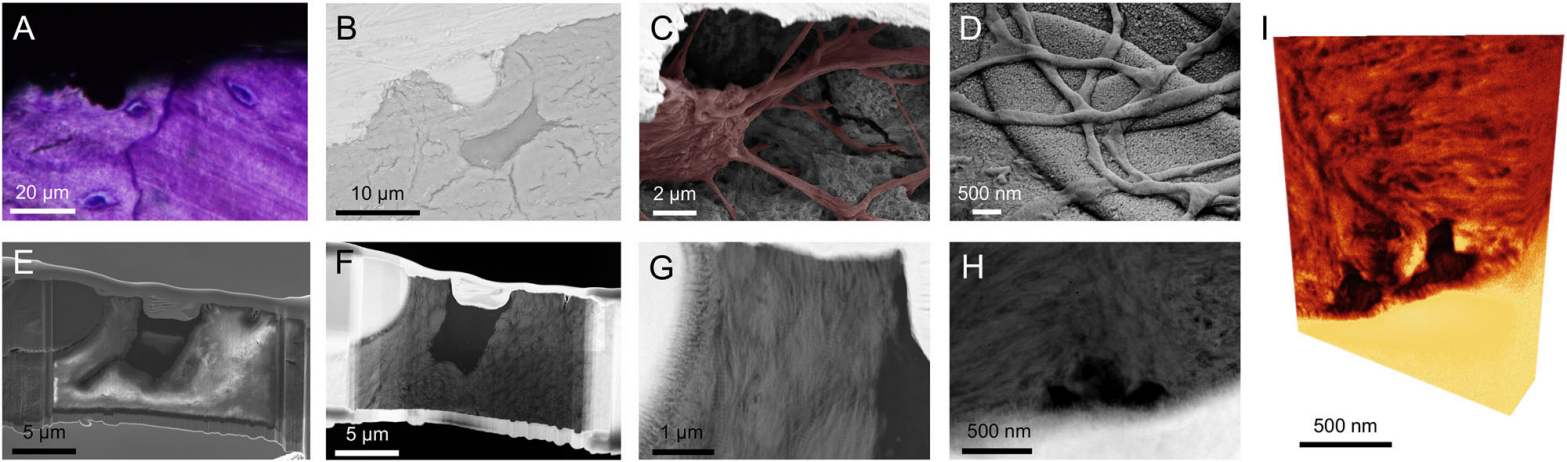
Osteocytes at the implant surface: The osteocyte connection to the implant surface. **a** Histological image of osteocytes close to the implant surface, the cell nuclei stained in blue. **b** BSE-SEM image of an osteocyte close to the implant surface. **c** Osteocyte close to the implant surface shown after resin cast etching where the canaliculi can be observed reaching the implant surface (Osteocyte and canaliculi highlighted in red). **d** Canaliculi making intimate contact with the implant surface, a top view showing the network of canaliculi. **e** FIB section during TEM sample preparation made across the osteocyte in **b**. A canaliculi is seen running in the 8 o’clock direction. **f** STEM image of the thin sample, the canaliculi was removed during the thinning process. **g** Closer view of the bone between the osteocyte lacuna and the implant surface showing a uniform directionality, indicating that the former osteoblast, now osteocyte produced the bone in a contact osteogenesis fashion. Collagen banding is observed perpendicular to the implant surface as well as the lacuna indicating collagen parallel to the surface, the morphology further indicate a mature bone with fibril bundles of 1–2 µm in diameter. **h** STEM image of canaliculi directly interfacing the implant surface. **i** Corresponding electron tomography volume rendering of **h** (Courtesy of Assistant Professor Kathryn Grandfield). Images reprinted and modified with permission from the American Chemical Society and Public Library of Science [27, 33]

The morphology of the tissue in association with the implant surface, bone lamellar orientation and alignment of osteocyte lacunae as well as the presence of osteocytes in the lacunae could be evaluated (Fig. 3). Bone remodeling is frequently observed both close to and at a distance from the implant surface where osteoblasts are often co-localized with osteoclasts as well as blood supply. The mineralization front (and therefore the direction of bone formation) and the embedment of differentiating osteocytes can be observed (Supplementary Figure 2) (Figs. 4 and 5).

### Raman spectroscopy

Raman spectroscopy is becoming increasingly used in the field of biomaterials, as a versatile tool requiring minimal sample preparation and is quick and minimally destructive. It has been used for material characterization [42], evaluation of in vitro formed extracellular matrix [43] as well as the molecular composition of bone tissue before [44] and after resin embedding [45] and is considered to give an assessment of the bone quality [46]. Site-specific analyses of interfaces such as natural bone interfaces, i.e., cement lines [47] or the bone-implant interface can be carried out [48, 49]. Confocal Raman imaging allows 2D and 3D mapping of composition at submicron resolution, and such data can be easily correlated with other imaging techniques. In composites such as bone, the relative amounts of the individual organic and inorganic constituents can be determined. Raman spectroscopy is particularly useful in identification of different calcium phosphate phases found in bone [50]. Furthermore, in experiments involving selective removal of tissue components for structural analysis, Raman spectroscopy can be used to identify and characterize the remaining component [51].

### Scanning electron microscopy (SEM)

SEM is a versatile and easy to use technique that enables high-resolution imaging in conjunction with chemical analyses. Different contrast phenomena can be used to highlight different aspects. The most frequently used are secondary electron (SE-SEM) and backscattered electron (BSE-SEM) modes, where the former gives contrast from the surface texture and the latter gives a *Z*-(atomic number) contrast. The *Z*-contrast is particularly useful for studying bone, since the degree of mineralization is directly resolved and could be calibrated using standard materials to determine the absolute wt. % calcium content [52]. In many ways, BSE-SEM provides a similar picture of the mineralized tissue as histology, where the morphology of the tissue, degree of mineralization and alignment of osteocytes could be directly visualized, in addition to details in remodeling zones with embedment of osteocytes and the granular appearance of the mineralization front (Supplementary Figure 2). Electron bombardment onto the sample surface induces charging artifacts, which are typically circumvented by the addition of a thin conductive coating. Another solution is the use of an environmental SEM operated at low vacuum with the presence of water vapor to minimize (or completely eliminate) the charging artifacts, without the need of an electrically conductive coating.

Secondary electron imaging generates contrast from surface irregularities and is hence used for evaluating osteocyte lacunae and the canaliculi network after resin cast etching [33] where the resin filled voids in bone could be highlighted, showing the intimate contact between the canalicular network and the implant surface as well as the interconnectivity of the LCN with the blood vessels and bone marrow [53].

In a successive manner, BSE-SEM is performed on polished surfaces without the use of a conductive coating. The sample is subsequently processed for resin cast etching, coated with a conductive coating for high vacuum SE-SEM, to be able to evaluate the same region of interest.

### Focused ion beam-scanning electron microscopy (FIB-SEM)

FIB-SEM is a useful tool in biomaterials research, where both 3D reconstructions could be performed using a serial slice-and-view (SSV) function. Much work has been done to study the ultrastructure of lamellar bone where imaging was performed through successive lamellae and reconstructing the lacuno-canalicular network, using a decalcification and staining protocol in order to visualize the collagen mesh [54] Without decalcification, the methodology has been used to visualize bone ingrowth in microporous titanium oxide, however without being able to resolve the unique ultrastructural characteristics of bone [55].

Another application of FIB-SEM is preparation of TEM samples. Thin lamellae or needle-shaped samples are cut out with sub-micrometer precision, readily cutting through both metal and undecalcified bone. The dimensions of the resulting sample are limited, and typically in the 25 × 1 µm range, while being able to obtain a thickness of roughly 100 nm. For more information on TEM sample preparation, the interested reader is referred elsewhere [56, 57].

### Transmission electron microscopy (TEM)

The TEM is a very powerful instrument with not only high magnifying power but also multiple in-built analytical techniques, such as diffraction, energy dispersive X-ray spectroscopy (EDS), and electron energy loss spectroscopy (EELS). For bone-implant interface analysis, during the 80 and 90s, the TEM was used mainly for imaging but no analytical techniques were implemented. The first chemical mapping was reported in 2006 [20] and much work has been done since. Furthermore, the use of scanning transmission electron microscopy (STEM) and the use of a nanoprobe allow elemental analysis with high spatial resolution as compared to the SEM where typically µm resolution is obtained. The contrast phenomena typically used are either bright-field TEM or high-angle annular dark field (HAADF-) STEM, where the latter gives *Z*-contrast and is preferred for the bone-implant interface.

The bone-implant interface has been shown to be composed of bundles of collagen fibrils running parallel to the implant surface in very close proximity to the oxide layer. In the case of nanostructured implant surfaces, apatite ingrowth into surface irregularities has been shown both by EDS [25, 28] and EELS [30, 33, 34]. The presence of features smaller than the thickness of the sample pose considerable difficulties in resolving the actual interface due an overlap in information, necessitating the use of high-resolution 3D imaging techniques.

For the osteocyte lacuno-canalicular network, as the sample is sectioned from only a top view, it is difficult to selectively have canaliculi in the sample, but due to their large presence in the bone, often transversely cut circular features (typically 200 nm, in agreement with canaliculi [23]) are visible in the TEM section, while part of osteocytes could be sectioned readily [33].

The structure and alignment of the tissue components, for example the size and orientation of apatite crystallites, can be evaluated, thus revealing details of the smallest hierarchical level of mineralized tissue in osseointegration [28]. The ultrastructural pattern of bone is highly dependent on the cutting direction, where mineralized collagen fibers may be cut longitudinally or transversally. In longitudinal sectioning, characteristic 67-nm striations are seen while holes and circular patterns are observed in transversal sectioning.

### Electron tomography (ET)

Electron tomography uses similar principle as micro-CT, where a rotation series of images is acquired, aligned, and reconstructed by back-projection. When performed in HAADF-STEM, similar contrast is obtained as in micro-CT and difficulties in reconstruction due to diffraction contrast as in regular TEM can be avoided. It has successfully been performed on the bone-implant interface enabling a greater understanding of apatite ingrowth into the nanostructured surface oxide layer [26, 30, 31, 33], however, limitations in the reconstruction due to lack of images originating from limited tilt angles creates artifacts in the images. The use of needle-shaped samples and special sample holders enables complete rotation and improved 3D reconstruction [34]. As image acquisition is performed with high *Z*-contrast, contrast based segmentation protocols similar to micro-CT can be applied, thus permitting quantification and enhanced visualization of collagen structure and orientation. Furthermore, with the use of needle-shaped sample, electron tomography could may be complemented by EELS tomography (chemical 3D mapping with nanometer resolution) and atom probe tomography (APT).

### Complementary analytical techniques

Additional complementary tools may be utilized at different stages of the sequential analytical approach and will further bridge the different length scales and bring new dimensions to the analysis.

**Fig. 6:**
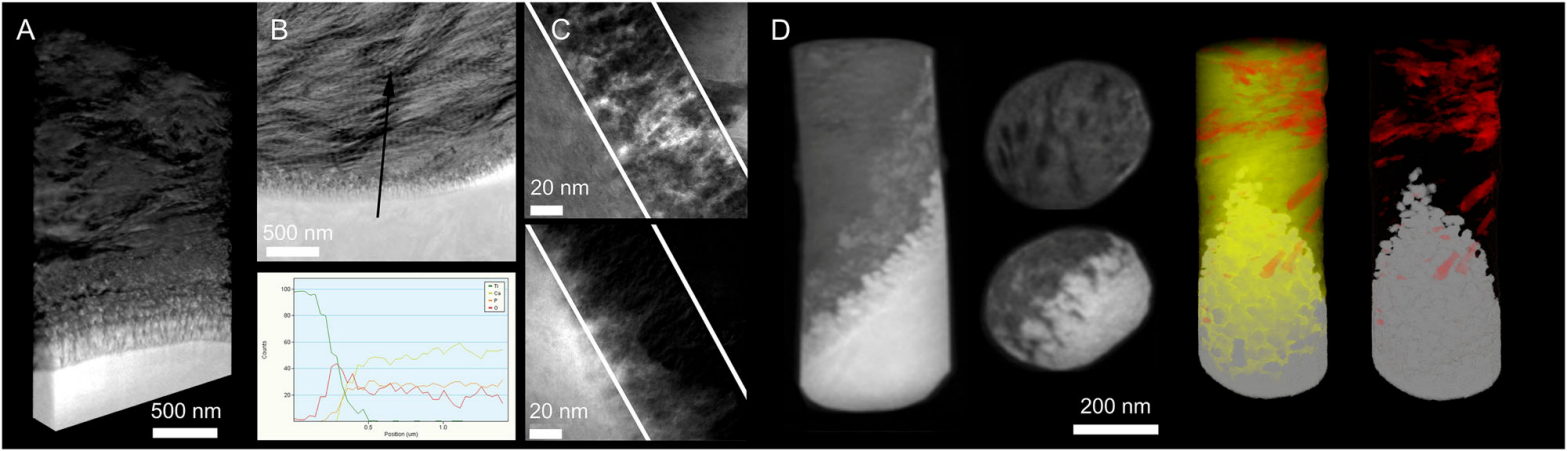
Nano-osseointegration: The nano-scale characteristics of the bone-implant interface could be visualized and analyzed using the transmission electron microscope. **a** A 3D rendering of the bone implant interface where the collagen bundles are observed parallel to the implant surface running in the plane of the section, collagen banding is observed perpendicular to the implant surface. The individual mineralized collagen fibrils which bundles up to make the bundles could be observed in the tomogram very close to the outermost surface of the oxide layer. **b** STEM image of the interface showing the typical collagen banding and parallel fashion of the collagen to the implant surface. Site specific chemical analysis by EDS across the interface show a zone of overlapping information indicating apatite formation in the nanostructures formed by the surface oxide. **c** TEM image of the interface with corresponding EFTEM image filtered for the calcium showing the ingrowth into the nanostructures. **d** A series of images of electron tomography of a needle-shaped implant where full rotation could be performed for improved reconstruction. In the perpendicular slices the bone structure with darker features (carbon rich collagen fibrils) and aligned apatite could be visualized while at the interface to the implant surface, the structure of the oxide layer with nanoscale features could be seen filled with apatite. Two 3D surface rendering of contrast-based segmented data set with implant (grey), collagen fibrils (red) and with/without apatite (yellow), showing the individual collagen fibrils seemingly being from two different collagen bundles (bundles typically in the range of 1–2 µm in diameter) with slightly different alignment, both however in a rather parallel to the implant surface. Images reprinted and modified with permission from the Royal Society (UK), the American Chemical Society and the Royal Society of Chemistry [26, 30, 33, 34]

Synchrotron radiation at large scale facilities is of high interest, where multiple different analyses could be carried out, using different sample sizes obtaining various fields of view and spatial resolution levels. Particularly for tomographic techniques, higher flux enables improved resolution and contrast than conventional methods [58]. Phase contrast tomography can be used for improved resolution and complement lab-based micro-CT, where improved resolution at the implant interface could be obtained, as well as observing and distinguish low contrast components in the bone tissue in 3D [59]. The average alignment of the mineralized collagen bundles and crystal thickness could be obtained in 2D [60, 61] and 3D [62] by scanning small-angle X-ray scattering (sSAXS) tomography at comparable low resolution with larger field of view. At higher resolution, the direction of individual collagen bundles can be investigated [63, 64] however in smaller sample volumes of a few hundreds of micrometers. 2D sSAXS has been applied to characterize bone healing around degradable metal implants [65, 66]. By ptychographic tomography, the osteocyte canaliculi network could be resolved in very small samples in the range of tens of micrometers [67]. It is possible to apply most of these techniques to plastic embedded samples at successive stages depending on their need for smaller samples sizes, and could hence be integrated in the proposed analytical approach. Following STEM tomography of needle-shaped samples preparing using the FIB (Fig. 6) [34], the tip can be thinned down further for EELS tomography and APT. APT is also becoming popular in the field of biomaterials [68] and bone research [69], where atomic resolution can be obtained in 3D. For further reading on high-resolution techniques, the interested reader is referred elsewhere [70].

## Concluding remarks

Through the use of correlative and complementary imaging and analytical techniques, osseointegration can be probed and evaluated at relevant hierarchical scales. The bone-implant interface shows an intimate contact between mineralized collagen bundles and the outermost surface of the oxide layer, while within the nanoscale surface features only bone mineral is found, thus creating a functionally graded interface. Osteocytes exhibit a direct contact with the implant surface via canaliculi that house their dendritic processes. Blood vessels are frequently found in close proximity to the implant surface either within the mineralized bone matrix or at regions of remodeling.

## Electronic supplementary material

Supplementary Information
