# Supplementary material for: A multiscale analytical approach to evaluate osseointegration
Source: J Mater Sci Mater Med. 2018 May 7;29(5):60. doi: 10.1007/s10856-018-6068-y (PMC5938308; doi:10.1007/s10856-018-6068-y)
Supplement: Supplementary file 1 — Supplementary Information [file 10856_2018_6068_MOESM1_ESM.pdf]

Electronic Supplementary Material

A multiscale analytical approach to evaluate osseointegration

Anders Palmquist [1,*]

[1] Department of Biomaterials, Sahlgrenska Academy at University of Gothenburg, Göteborg, Sweden;

* Corresponding author: Anders Palmquist, Department of Biomaterials, Box 412; 405 30 Göteborg; Sweden; anders.palmquist@biomaterials.gu.se

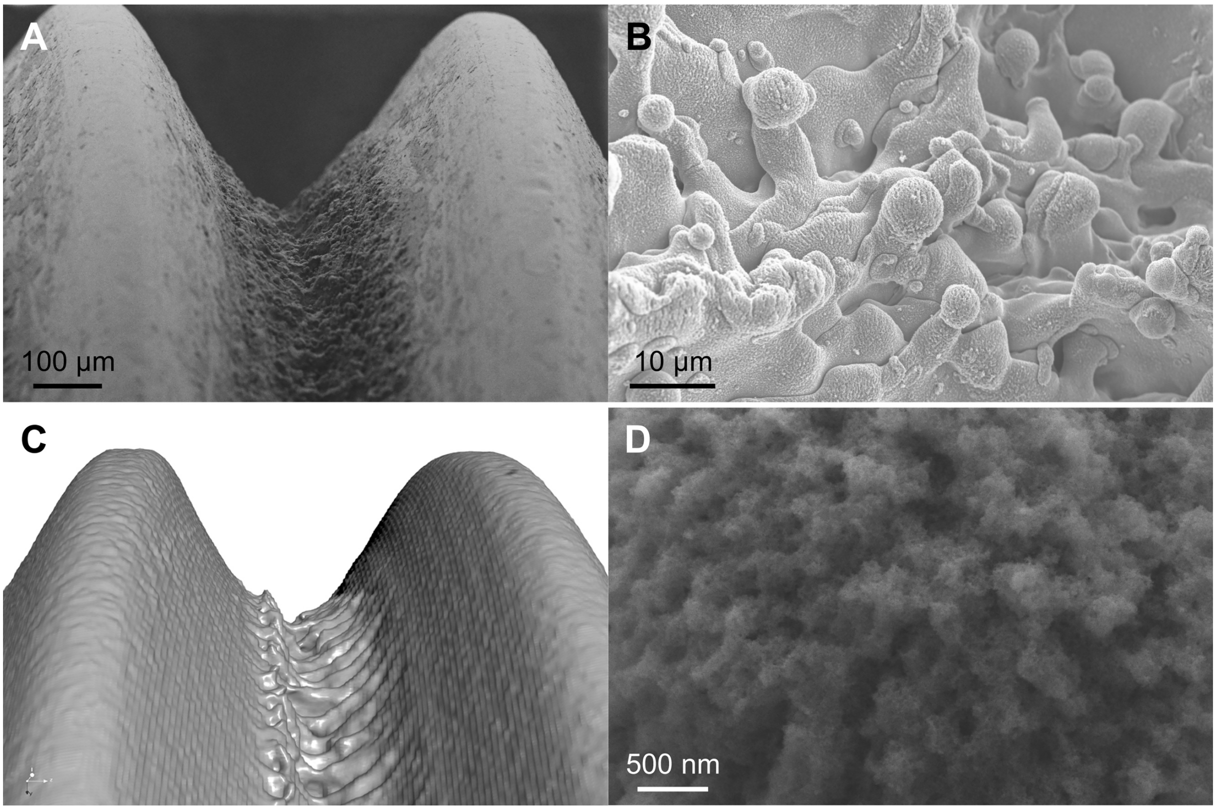

Supplementary Figure 1: Typical surface images of the implant type used to exemplify the bone-implant interface with the different techniques. A) Low resolution SEM image of the surface. B) The surface micrometer features. C) A volume rendering of the implant from micro-CT. D) The nanoscale features. Reprinted with permission from Elsevier [1]

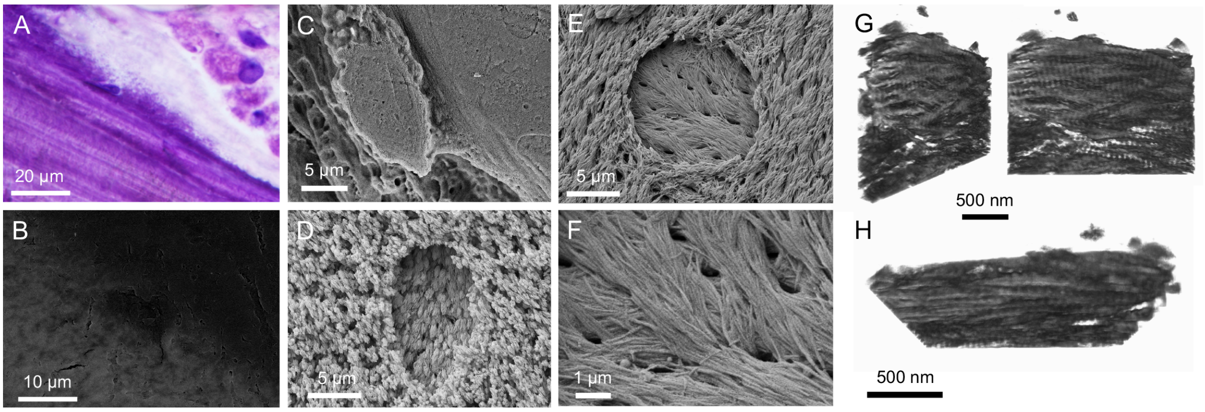

Supplementary Figure 2: Images of osteocyte embedment during bone formation and the mature bone structure interfacing the lacuna after dynamic bone formation. A) Histological figure showing the same remodeling zone as in Figure 4 showing the progressing mineralization of the tissue as granular appearance. B) BSE-SEM of a similar osteocyte embedment where a decreasing contrast is clearly visible toward the bone formation front, indicating the progressive mineralization, also here a granular appearance is seen. C) Recently fully embedded osteocyte after resin cast etching, the ultrastructure of the cell could be observed with the cell nucleus. D) A deproteinized endosteal bone surface showing the lacuna where the osteocyte was being embedded in and the surrounding bone during mineralization. A granular appearance of the apatite is seen in the mineralizing tissue. E) Similar view as in D, however at a location where the bone mineralization has been completed at the endosteal surface, interesting is the structure and alignment of the bone at the floor of the lacuna, showing the collagen fibril bundles with a typical diameter of 1-2 µm as well as the holes in-between the bundles indicating the canaliculi from the lacuna. F) Higher magnification showing the floor of the lacuna, showing the rope-like structure of the collagen fibril bundles composed of approximately 50 nm wide collagen fibrils where most of the fibrils are aligned with the bundle while some deviates. Furthermore, the shift in bundle direction in the deeper layer is clearly seen indicating the rotating direction. G) Electron tomograms of the bone facing the osteocyte lacuna, showing similar bundle structure and fibril sizes as in the deproteinized sample. The collagen banding is seen with the 67 nm repetition. H) Tilted view of the tomogram showing the top surface, similar to in F. Images reproduced and modified with permission from Springer Nature and the American Chemical Society [2-4]

References

1. Shah FA, Nilson B, Branemark R, Thomsen P, Palmquist A. The bone-implant interface - nanoscale analysis of clinically retrieved dental implants. Nanomedicine. 2014;10(8):1729-37. doi:10.1016/j.nano.2014.05.015.

2. Shah FA, Palmquist A. Evidence that Osteocytes in Autogenous Bone Fragments can Repair Disrupted Canalicular Networks and Connect with Osteocytes in de novo Formed Bone on the Fragment Surface. Calcif Tissue Int. 2017;101(3):321-7. doi:10.1007/s00223-017-0283-2.

3. Shah FA, Wang X, Thomsen P, Grandfield K, Palmquist A. High-resolution visualisation of the osteocyte lacuno-canalicular network juxtaposed to the surface of nanotextured titanium implants in human. Acs Biomater Sci Eng. 2015;1(5):305-13.

4. Shah FA, Zanghellini E, Matic A, Thomsen P, Palmquist A. The Orientation of Nanoscale Apatite Platelets in Relation to Osteoblastic-Osteocyte Lacunae on Trabecular Bone Surface. Calcif Tissue Int. 2016;98(2):193-205. doi:10.1007/s00223-015-0072-8.
